# Evolving Treatment Strategies for Neuropathic Pain: A Narrative Review

**DOI:** 10.3390/medicina61061063

**Published:** 2025-06-10

**Authors:** Alan D. Kaye, Grace Armistead, Lane S. Amedio, Mills E. Manthei, Shahab Ahmadzadeh, Brian Bernhardt, Sahar Shekoohi

**Affiliations:** 1 Departments of Anesthesiology and Pharmacology, Toxicology and Neurosciences, Louisiana State University Health Sciences Center Shreveport, Shreveport, LA 71103, USA; 2 School of Medicine, Louisiana State University Health Sciences Center Shreveport, Shreveport, LA 71103, USAmem004@lsuhs.edu (M.E.M.); 3 Department of Anesthesiology, Louisiana State University Health Sciences Center Shreveport, Shreveport, LA 71103, USA

**Keywords:** chronic neuropathic pain, nerve injury, neuropathic pain, nociception, peripheral nerve injury, neuropathy

## Abstract

Neuropathic pain resulting from injury to the somatosensory nervous system affects approximately 6.9–10% of the general population and significantly impacts quality of life. Common presentations include burning, stabbing, tingling, or electrical sensations, occurring spontaneously or through hyperalgesia or allodynia. Treatment approaches follow a tiered system. First-line therapies include gabapentinoids (e.g., gabapentin, pregabalin), which target voltage-gated calcium channels; tricyclic antidepressants (e.g., amitriptyline, nortriptyline); and serotonin-norepinephrine reuptake inhibitors such as duloxetine. Second-line options encompass topical agents (e.g., 5% lidocaine, 8% capsaicin), opioid-like medications (e.g., tramadol, tapentadol), and adjunctive therapies including psychological therapies and lifestyle interventions. For refractory cases, third-line treatments include NMDA receptor antagonists (e.g., ketamine, dextromethorphan), cannabinoids, and botulinum toxin type A, though these have more limited clinical evidence. Procedural interventions such as spinal cord stimulation and transcutaneous electrical nerve stimulation provide alternatives when pharmacological approaches fail. Despite advances in treatment options, many patients remain undertreated, highlighting the need for individualized, multimodal approaches and continued research into the complex pathophysiology of neuropathic pain conditions.

## 1. Introduction

**Figure 1:**
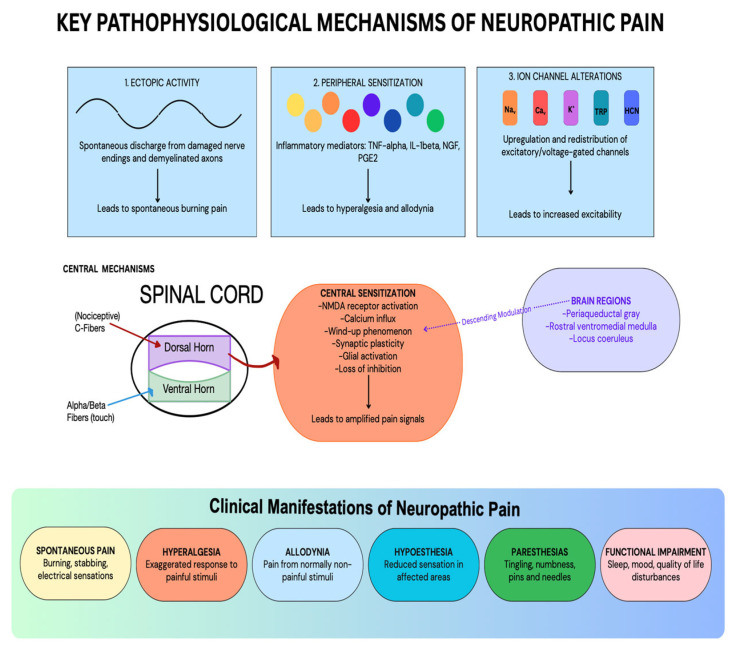
Key Pathophysiological Mechanisms of Neuropathic Pain [2,5,6,7].

Neuropathic pain, defined by the International Association for the Study of Pain (IASP) as “pain caused by a lesion or disease of the somatosensory nervous system,” represents a significant healthcare challenge affecting 6.9–10% of the general population [1,2]. Unlike nociceptive pain, which serves a protective function, neuropathic pain often becomes chronic, losing its adaptive value and transforming into a self-perpetuating pathological symptom [3,4]. The pathophysiology of neuropathic pain is remarkably complex, involving multiple mechanisms including ectopic activity in damaged nerves, peripheral and central sensitization, ion channel alterations, and imbalances between excitatory and inhibitory signaling pathways [5,6]. These changes result in disordered transmission of sensory signals into the spinal cord and the brain, which explains why neuropathic pain is often refractory to conventional analgesics and requires specialized treatment approaches [7]. These mechanisms and their clinical manifestations may be visualized below in Figure 1 [2,5,6,7]. This narrative review aims to synthesize current knowledge on neuropathic pain pathophysiology and treatment options to provide clinicians with a comprehensive framework for managing this challenging condition.

Diagnosing neuropathic pain involves a comprehensive assessment of sensory symptoms and signs, with patients typically describing pain as burning, stabbing, electrifying, or tingling [3,8]. Various screening tools such as the Douleur Neuropathique en 4 Questions (DN4), the I-DN4 (self-administered DN4), Leeds Assessment of Neuropathic Symptoms and Signs (LANSS), painDETECT, and the Neuropathic Pain Symptom Inventory (NPSI) have been developed to facilitate diagnosis and quantify neuropathic symptoms [2,7,9]. Among these methods, the strongest recommendations are made for the DN4, I-DN4, and LANSS by the Joint European Academy of Neurology [9]. Confirmatory tests can enhance diagnostic certainty, including quantitative sensory testing, neurophysiological techniques, skin biopsy, and neuroimaging [7,10].

Treatment of neuropathic pain requires a multimodal approach. This narrative review will follow a tiered approach examining first-line, second-line, and third-line treatment options. First-line pharmacotherapy includes gabapentinoids (e.g., gabapentin, pregabalin), tricyclic antidepressants (e.g., amitriptyline, nortriptyline), and serotonin-norepinephrine reuptake inhibitors (e.g., duloxetine, venlafaxine) [8,10]. Second-line options comprise topical agents (e.g., lidocaine patches, capsaicin creams, and patches) and tramadol, with strong opioids reserved as third-line therapies for refractory cases [3,7]. Despite these options, the management of neuropathic pain remains challenging, with fewer than 50% of patients achieving satisfactory relief [10]. This has led to increased interest in interventional therapies, such as nerve blocks, neuromodulation techniques including spinal cord and peripheral nerve stimulation, and intrathecal drug delivery for treatment-resistant cases [3,11]. Additionally, non-pharmacological approaches, including physical therapy, psychological interventions, and lifestyle modifications, are important components of a comprehensive treatment plan [4,8].

The complexity of neuropathic pain necessitates a personalized approach to treatment. Emerging strategies include targeting treatments based on sensory phenotypes rather than etiology alone, which may improve therapeutic outcomes [5,12]. Recognizing the significant impact of neuropathic pain on quality of life, interdisciplinary management that addresses both the physical and psychosocial dimensions of pain is increasingly recommended [4].

Therefore, the present investigation examines current evidence regarding pharmacological and non-pharmacological approaches to managing neuropathic pain, including first-line treatments such as anticonvulsants, antidepressants, and topical agents, as well as emerging therapies and interventional techniques for refractory cases. The review also explores special considerations for different populations and provides insights into future directions in neuropathic pain management.

## 2. First-Line Treatments for Neuropathic Pain

When managing neuropathic pain, the primary objective is to alleviate patient suffering while enhancing functional outcomes. First-line treatments are typically selected based on proven efficacy in clinical trials and supported by patient-reported outcomes. The goal is to reduce pain intensity, improve functionality, and enhance overall quality of life. Although some holistic approaches have shown benefits in selected patients, most first-line therapies are pharmacological.

Gabapentinoids are often considered the cornerstone of first-line treatment for neuropathic pain. These agents act by targeting the α2δ subunit of voltage-gated calcium channels, leading to decreased neuronal excitability [8]. In this regard, efficacy has been demonstrated in conditions such as diabetic neuropathy, postherpetic neuralgia, and spinal cord injuries. Gabapentin has a half-life of 5 to 7 hours and is commonly prescribed in divided dosages targeting a total of 1800 mg per day. Extended release gastroretentive gabapentin, e.g., GRALISE^®^, and the prodrug of gabapentin, HORIZANT^®^ (e.g., gabapentin enacarbil), have fewer side effects and can be taken once daily after dinner. Pregabalin, a next-generation gabapentinoid with more predictable absorption and faster onset, is typically prescribed between 300 and 600 mg per day and also has a controlled-release formulation, Lyrica CR^®^ [8]. Randomized controlled trials have validated the effectiveness of gabapentin, particularly in postherpetic neuralgia and diabetic neuropathy, leading to FDA approval for both gabapentin and pregabalin [8]. Although frequently used, treatment with immediate release gabapentinoids is not without drawbacks. Some commonly reported side effects include sedation, weight gain, dizziness, fatigue, suicidal thoughts, depression, and cognitive impairment, and there is a recognized risk of misuse, respiratory depression, and abuse [8].

Another key group of medications used as first-line agents is tricyclic antidepressants (TCAs). These drugs help modulate pain pathways through inhibition of serotonin and norepinephrine reuptake [8]. Although found to be effective in treating neuropathic pain, side effects, such as anticholinergic symptoms, can limit tolerability [7]. Tricyclic antidepressants, e.g., amitriptyline and nortriptyline, have been indicated in the treatment of cryptogenic sensory polyneuropathy [8]. Dosing typically falls within a range of 25–150 mg/day [8].

For patients who may not tolerate tricyclic antidepressants, serotonin norepinephrine reuptake inhibitors (SNRIs) may be considered as an alternative. SNRIs such as duloxetine are commonly selected, related to favorable side effect profiles in comparison to other neuropathic pain treatments [8]. Their mechanism of action involves preventing serotonin and norepinephrine reuptake, thereby enhancing descending pain inhibitory pathways [7]. SNRI treatment plans are commonly seen when managing diabetic neuropathy [8].

Choosing among first-line agents such as gabapentinoids, tricyclic antidepressants, and serotonin norepinephrine reuptake inhibitors often requires an individualized approach based on patient-specific factors such as age, comorbidities, and any prior treatment successes or failures [7,8] (Table 1). In some cases, combination treatment may be considered when monotherapy does not provide sufficient relief to the patient [7,8].

## 3. Second-Line Treatments for Neuropathic Pain

Although many first-line treatments are frequently used with success, second-line therapies represent an important group of medications available for those needing alternatives. Topical therapies represent an important class of second-line medications for neuropathic pain. These include both lidocaine and capsaicin. The 5% lidocaine patches act by blocking sodium channels, and high-dose capsaicin works through TRPV1 receptor desensitization [8]. Capsaicin patches are typically 8% and decrease the sensitivity of nociceptors [8]. Capsaicin cream formulations are also utilized and have been shown to be effective, and are commonly applied every three months for diabetic neuropathic pain states.

Although considered highly effective in certain cases, opioids are generally classified as second-line related to their high risk for dependence and unfavorable side effect profile. Medications such as tramadol or tapentadol may be preferred due to dual action on opioid receptors and serotonin/norepinephrine pathways [3,8]. The primary mechanism of action involves μ opioid receptor agonism alongside some SNRI activity [3,8].

Complementary approaches can augment pharmacological management and address broader aspects related to chronic pain. Some non-pharmacological strategies include physical therapy, psychological therapies, and lifestyle interventions focused on improving sleep and physical activity [8]. Psychological approaches include cognitive behavioral therapy, acceptance and commitment therapy, and pain reprocessing therapy [8].

In severe neuropathic pain that is unresponsive to first- and second-line therapy, interventional and surgical procedures may be considered [3,7]. These options include neuromodulation via the spinal cord, peripheral nerve, and transcutaneous electrical nerve stimulation. [3,7]. Spinal cord peripheral nerve stimulation requires a trial, and then, a surgical procedure to implant electrodes in the epidural space of the spinal cord or along the affected peripheral nerve is performed [3,7]. This allows for the delivery of electrical impulses directly to the spinal cord or at the level of the affected peripheral nerve to modify pain perception at the central or peripheral nerve level. Transcutaneous electrical nerve stimulation (TENS) is a noninvasive procedure that delivers low-voltage electrical currents through surface electrodes to stimulate superficial nerves and modulate pain pathways [3,7]. TENS is frequently used in localized pain, as it is portable, inexpensive, and can be used at home or in outpatient settings [3,7].

Additional therapies include intrathecal drug delivery systems and surgical decompression, if applicable [3]. However, despite the numerous therapies that have been developed, many patients remain undertreated, which highlights the need for increased understanding of treatment options, ongoing research, and personalized treatment strategies [7] (Table 2).

## 4. Third-Line Treatments for Neuropathic Pain

Third-line treatments for neuropathic pain are typically reserved for patients who have not achieved adequate relief or have experienced intolerable side effects from first and second-line therapies. These agents often have more limited or conflicting evidence, narrower indications, or significant adverse effect profiles. Nonetheless, they target unique mechanisms in the neurobiology of pain and can offer therapeutic benefits in select clinical contexts.

One group of third-line agents includes N-methyl-D-aspartate (NMDA) receptor antagonists, most notably ketamine and dextromethorphan. NMDA receptors, activated by glutamate, play a pivotal role in central sensitization, a process that amplifies pain perception following peripheral nerve injury [13]. Enhanced NMDA receptor activity facilitates calcium influx, promoting synaptic potentiation and increased neuronal excitability [13].

Ketamine, a noncompetitive NMDA receptor antagonist, disrupts this pathway by blocking calcium entry, thereby dampening nociceptive transmission. Sub-anesthetic doses of ketamine between 75 to 475 mg are typically delivered via intravenous infusion and have demonstrated efficacy in conditions such as complex regional pain syndrome (CRPS), diabetic neuropathy, and postherpetic neuralgia [13]. Dosing is based on pre-clinical studies. While analgesia can be rapid, the duration of relief is variable, often requiring repeated treatments. Given the potential for adverse effects, including psychotomimetic symptoms, transient hypertension, hepatotoxicity, apnea, sedation, hypersalivation, hallucinations, dizziness, and drowsiness, its administration is typically restricted to closely supervised environments such as inpatient pain management units or specialized clinical settings [13].

Dextromethorphan is an orally administered N-methyl-D-aspartate receptor (NMDAR) antagonist that, while exhibiting lower potency compared to other agents in its class, offers a convenient and non-invasive route of administration [13]. In addition to NMDAR antagonism, it modulates sigma-1 receptors and inhibits α3/β4 nicotinic acetylcholine receptors, contributing to its potential analgesic and neuroprotective effects [13]. Preclinical studies have demonstrated efficacy in reversing tactile allodynia and thermal hyperalgesia, with effective doses reported at 20 mg/kg orally and 10 nmol intrathecally [13]. However, clinical application in neuropathic pain remains limited due to poor and inconsistent bioavailability, as well as variable efficacy across patient populations. Common adverse effects include drowsiness, dizziness, confusion, gastrointestinal discomfort, and, in some cases, hallucinations and motor disturbances [13]. As a result, dextromethorphan is more commonly utilized as an adjunctive therapy rather than a standalone treatment, particularly when combined with agents such as gabapentin or opioids, which may enhance its antiallodynic effects.

Another class of emerging interest in third-line neuropathic pain management is cannabinoids, including both plant-derived (e.g., phytocannabinoids) and synthetic analogs. The two primary constituents are tetrahydrocannabinol (THC) and cannabidiol (CBD), which exert effects primarily via cannabinoid receptor type 1 (CB1) and type 2 (CB2) [14]. CB1 receptors are predominantly expressed in the central nervous system, where they modulate neurotransmitter release, while CB2 receptors are found on immune cells and peripheral tissues, influencing inflammatory responses [15].

Activation of CB1 receptors reduces presynaptic calcium influx and blocks the release of excitatory neurotransmitters, e.g., glutamate and substance P, contributing to reduced central sensitization [15]. CB2 receptor activity may modulate immune cell function and neuroinflammation, which are increasingly recognized contributors to neuropathic pain pathophysiology [15].

Clinical trials have demonstrated that cannabinoids can modestly reduce peripheral neuropathic pain, with 79% of randomized controlled trials reporting statistically significant improvements. A meta-analysis showed an average reduction of 0.67 points on a 10-point pain scale compared to the placebo [14]. However, variability in cannabinoid formulations, delivery methods, and dosing regimens complicates interpretation. Flexible dosing schedules appear to improve tolerability, suggesting a benefit to individualized titration. Consensus recommendations for routine dosing published by Bhaskar et al. suggest starting with 5 mg CBD-predominant oral preparation twice daily and up-titrating 10 mg per day up to 40 mg of CBD per day. The maximum allowed amount of THC in CBD-predominant strains is 1:10 (THC:CBD), and starting with CBD-predominant preparations has a lower risk for undesirable side effects [16]. Side effects such as dry mouth, fatigue, sedation, and cognitive changes are generally mild and transient [14]. Related to concerns about psychoactive effects and inconsistent efficacy, cannabinoids are not considered first-line treatments but may be appropriate in refractory cases or when standard therapies are contraindicated, particularly in regulated medical formulations.

Botulinum toxin type A (BoNT-A) has also emerged as a targeted peripheral approach to neuropathic pain. BoNT-A is a neurotoxin that cleaves SNAP-25, a key SNARE protein involved in synaptic vesicle fusion and neurotransmitter release [17]. By inhibiting the exocytosis of glutamate, substance P, and calcitonin gene-related peptide (CGRP) from nociceptive neurons, BoNT-A attenuates peripheral and, indirectly, central sensitization [17].

Botulinum toxin type A (BoNT-A) is administered through localized subcutaneous or intradermal injections at the site of maximal pain and has shown clinical benefit in conditions such as postherpetic neuralgia, diabetic neuropathy, and trigeminal neuralgia [17]. Its side effect profile is generally favorable, with the most commonly reported adverse events including transient injection site discomfort, mild muscle weakness, and localized erythema [18]. Despite its therapeutic potential, the use of BoNT-A is limited by the need for specialized training, the requirement for repeated administration, and high treatment costs, which constrain its accessibility in routine clinical practice.

Third-line treatments for neuropathic pain, including NMDA receptor antagonists, cannabinoids, and botulinum toxin A, offer diverse strategies to address symptoms. While not universally applicable, they may serve as valuable tools in individualized pain management plans when conventional options have been exhausted (Table 3).

## 5. Non-Pharmacologic and Procedural Treatments for Neuropathic Pain

While pharmacologic interventions form the cornerstone of neuropathic pain management, a significant subset of patients experience inadequate relief or encounter intolerable adverse effects. Non-pharmacologic and procedural therapies play a critical adjunctive or alternative role, particularly in complex or refractory cases. These strategies target peripheral, central, and psychosocial mechanisms implicated in chronic neuropathic pain and are increasingly emphasized in multidisciplinary treatment plans [4].

Neuromodulation encompasses a range of procedural interventions that modulate aberrant nociceptive signaling at the spinal or peripheral level. These techniques are particularly valuable in the management of neuropathic pain, offering alternative pathways for analgesia when conventional pharmacologic strategies prove insufficient. By employing electrical stimulation or targeted delivery of therapeutic agents, neuromodulation aims to alter neural circuit function and restore a more physiologic balance in pain-processing networks [19].

Spinal cord stimulation (SCS) is a well-established invasive neuromodulatory technique involving the epidural implantation of electrodes that deliver patterned electrical impulses to the dorsal columns of the spinal cord [19]. The primary mechanism of action is thought to involve the activation of large-diameter Aβ fibers, which exert inhibitory control over smaller nociceptive C-fibers through segmental mechanisms, as described in the Gate Control Theory [20]. This results in a reduction of pain transmission at the spinal level and subsequent attenuation of pain perception. Clinical trials and longitudinal studies have demonstrated significant reductions in pain intensity and improvements in quality of life in patients with complex regional pain syndrome (CRPS), failed back surgery syndrome, and certain forms of peripheral neuropathy [20].

SCS carries a range of procedural and long-term risks. Common complications include lead migration, which can reduce the efficacy of stimulation and may require revision surgery [19]. Infection is another concern, particularly at the surgical site or around the implanted pulse generator, with some cases necessitating device explantation [19]. In rare instances, SCS may result in neurological injury, including spinal cord or nerve root damage, particularly during lead placement [19]. Patients may also experience unwanted stimulation or loss of efficacy over time related to physiological adaptation or changes in pain patterns.

A thorough preoperative evaluation, spinal cord stimulation trialing with leads placed for a week with positive results, careful surgical permanent implantation technique, and close postoperative monitoring are essential to mitigate these risks.

Peripheral nerve stimulation has been described throughout the body with a variety of commercial lead options. As with SCS, a peripheral nerve trial, with leads placed for a week with positive results, careful surgical permanent implantation technique, and close postoperative monitoring, is critical to ensure the best outcome for these patients.

Similarly, transcutaneous electrical nerve stimulation (TENS) is a non-invasive analgesic modality that delivers low-voltage electrical currents through surface electrodes to modulate peripheral and central pain pathways. Its mechanism of action is multifactorial, primarily involving stimulation of large-diameter Aβ fibers that inhibit nociceptive C-fiber input at the spinal level, consistent with the Gate Control Theory [21]. TENS may also reduce central sensitization, a key contributor to chronic neuropathic pain, by decreasing dorsal horn neuron transmission [21] (Table 4).

## 6. Discussion

The tiered treatment approach outlined in the present investigation reflects an evolving understanding of pain mechanisms and the need for individualized management strategies. While first-line treatments such as gabapentinoids, tricyclic antidepressants, and SNRIs remain the foundation of pharmacological management, efficacy varies considerably among patients with different neuropathic pain conditions.

The emergence of topical agents as second-line options offers localized treatment with potentially fewer systemic side effects, making them particularly valuable for elderly patients or those with multiple comorbidities [8]. However, the clinical utility of opioid-like medications such as tramadol and tapentadol remains constrained by concerns regarding dependence and adverse effects, necessitating careful patient selection and monitoring [3,8].

Third-line treatments, including NMDA receptor antagonists, cannabinoids, and botulinum toxin A, represent important alternatives for refractory cases, though their implementation is often limited by restricted evidence, adverse effect profiles, or practical considerations such as administration requirements and cost [14,15]. Ketamine, for instance, has shown promise in conditions such as complex regional pain syndrome but requires specialized administration settings related to potential psychotomimetic effects [15]. Similarly, while cannabinoids have demonstrated modest efficacy in certain neuropathic conditions, variability in formulations and regulatory considerations affect their widespread adoption [14].

The increasing recognition of neuromodulation techniques such as spinal cord stimulation, peripheral nerve stimulation, and transcutaneous electrical nerve stimulation highlights a shift toward multimodal approaches that target multiple pain pathways simultaneously [19,21]. These interventions can provide significant relief when pharmacological strategies have been exhausted, though considerations regarding patient selection, procedural risks, and cost-effectiveness remain pertinent [20].

Despite these advances, a substantial proportion of patients with neuropathic pain experience inadequate relief or intolerable side effects with first- and second-line treatments [7]. This therapeutic gap underscores the need for better education of patients and healthcare providers of the interventional pain procedures currently available, continued research into novel analgesic targets, and personalized treatment algorithms that consider individual pain mechanisms, genetic factors, and psychosocial contexts.

Newly emerging research in the astrocytes of rat models has identified several potential targets for novel analgesic therapies. S100A4 is a Ca^2+^-binding protein known to induce inflammation via its activation of cytokines and pro-inflammatory pathways. Xue et al. identified that the TLR4/NF-κB signaling pathway is a downstream regulator of S100A4 activation, and that specific depletion of this pathway suppresses deleterious A1 astrocyte activation and further attenuates the development and maintenance of neuropathic pain [22]. In short, this research provides a potentially promising new avenue for directed therapies in patients who suffer from neuropathic pain.

In the future, pain medicine would benefit from larger, well-designed clinical trials that incorporate mechanism-based patient stratification and meaningful functional outcomes beyond pain intensity ratings. Additionally, greater emphasis on integrative approaches that combine pharmacological interventions with physical rehabilitation, psychological therapies, and lifestyle modifications may enhance overall treatment efficacy [4]. Such comprehensive strategies are particularly relevant given the complex interplay between neuropathic pain, mood disorders, and functional impairment that characterizes the clinical experience of affected individuals.

## 7. Conclusions

Neuropathic pain management continues to evolve as our understanding of underlying pathophysiological mechanisms expands. The present investigation examined current evidence for pharmacological and non-pharmacological approaches, highlighting the strengths and limitations of available interventions within a tiered treatment framework. The prevalence of neuropathic pain, which affects 6.9–10% of the general population, underscores the significant public health burden and the imperative for effective management strategies [3,8].

First-line therapies, including gabapentinoids, tricyclic antidepressants, and SNRIs, remain foundational in the management approach, offering clinically meaningful relief for many patients [8]. The efficacy of these agents has been well-documented across various neuropathic conditions, particularly diabetic neuropathy and postherpetic neuralgia [3]. However, side effect profiles and variable patient responses necessitate careful consideration of individual patient factors when selecting initial treatment approaches [8].

For patients with inadequate response to first-line interventions, second-line options such as topical agents, opioid-like medications, and complementary approaches provide important alternatives [8]. The utility of topical lidocaine and capsaicin in providing localized relief with minimal systemic absorption represents a significant advantage in selected patients [8]. Meanwhile, the judicious use of tramadol and tapentadol balances analgesic efficacy against concerns regarding dependence and adverse effects [3].

Third-line treatments, including NMDA receptor antagonists, cannabinoids, and botulinum toxin A, offer therapeutic options for refractory cases, albeit with more limited evidence and specific administration requirements [14,15]. These interventions target distinct mechanisms in the pain pathway and may be particularly valuable when conventional approaches have been exhausted.

Procedural interventions, such as spinal cord stimulation, peripheral nerve stimulation, and transcutaneous electrical nerve stimulation, have demonstrated efficacy in modulating pain signaling pathways and improving quality of life in selected patients [21,22]. These neuromodulatory approaches represent an important component of the comprehensive management strategy, particularly for those with refractory symptoms.

Despite significant advances in treatment options, many patients with neuropathic pain remain undertreated, highlighting the need for improved education of both patients and healthcare providers, continued research, and innovation [7]. In this regard, technical advances in both spinal cord stimulation and peripheral nerve stimulation have improved the success rates and tolerability of these devices for neuropathic pain states [8,15,22]. Future directions should focus on identifying novel therapeutic targets, developing mechanism-based treatment algorithms, and implementing personalized approaches that consider the unique clinical, genetic, and psychosocial factors influencing pain experience.

In conclusion, optimal management of neuropathic pain requires a multidisciplinary approach that integrates pharmacological (first-line to third-line treatment) and non-pharmacological interventions within a holistic, patient-centered framework, which some specialized hospitals offer [23]. By addressing the complex pathophysiology of neuropathic states through targeted, multimodal strategies, clinicians can work toward improving outcomes and enhancing the quality of life for those affected individuals worldwide.

## Figures and Tables

**Table 1:** First-Line Treatments for Neuropathic Pain.

Treatment Class	Specific Medications	Dosage	Mechanism of Action	Indications	Side Effects
**Gabapentinoids**	Gabapentin	1200–3600 mg/day	Target α2δ subunit of voltage-gated calcium channels, decreasing neuronal excitability	Diabetic neuropathy, postherpetic neuralgia, spinal cord injuries	Sedation, dizziness, risk of misuse and abuse
**Gabapentinoids**	Pregabalin	300–600 mg/day	Same as gabapentin, with more predictable absorption and faster onset	Same as gabapentin	Similar to gabapentin
**Tricyclic Antidepressants (TCAs)**	Amitriptyline, Nortriptyline	25–150 mg/day	Inhibit serotonin and norepinephrine reuptake	Cryptogenic sensory polyneuropathy	Anticholinergic symptoms
**Serotonin-Norepinephrine Reuptake Inhibitors (SNRIs)**	Duloxetine, Venlafaxine		Prevent reuptake of serotonin and norepinephrine	Diabetic neuropathy	More favorable side effect profile than TCAs

**Table 2:** Second-Line Treatments for Neuropathic Pain.

Treatment Class	Specific Medications/Approaches	Mechanism of Action	Notes
**Topical Agents**	5% Lidocaine patches and creams	Block sodium channels	Localized treatment with fewer systemic side effects
	8% Capsaicin patches and creams	TRPV1 receptor desensitization	Decreases sensitivity of nociceptors
**Opioid-like Medications**	Tramadol, Tapentadol	μ-opioid receptor agonism with SNRI activity	Generally avoided as first-line due to high risk for dependency
**Complementary Approaches**	Physical therapy	Various physical modalities	Augments pharmacological management
**Complementary Approaches**	Psychological therapies (CBT, ACT, pain reprocessing)	Address cognitive and behavioral aspects of pain	Addresses broader aspects of chronic pain
**Complementary Approaches**	Lifestyle interventions	Improve sleep and physical activity	Supports overall pain management

**Table 3:** Third-Line Treatment Options for Neuropathic Pain.

Treatment Class	Specific Medications	Administration	Mechanism of Action	Clinical Applications	Limitations
**NMDA Receptor Antagonists**	Ketamine	IV infusion, 75–475 mg (sub-anesthetic)	Blocks calcium entry by antagonizing NMDA receptors	CRPS, diabetic neuropathy, postherpetic neuralgia	Psychotomimetic symptoms, hypertension, hepatotoxicity; requires supervised settings
	Dextromethorphan	Oral, 20 mg/kg; or intrathecal, 10 nmol	NMDAR antagonism, modulates sigma-1 receptors, inhibits nicotinic acetylcholine receptors	Adjunctive therapy	Poor bioavailability, variable efficacy
**Cannabinoids**	THC, CBD	Various	Activation of CB1 (CNS) and CB2 (immune cells) receptors	Modest reduction in peripheral neuropathic pain	Variable formulations, regulatory issues, psychoactive effects
**Botulinum Toxin Type A**	BoNT-A	Localized subcutaneous/intradermal injections	Inhibits exocytosis of pain neurotransmitters	Postherpetic neuralgia, diabetic neuropathy, trigeminal neuralgia	Requires specialized training, repeated administrations, high cost

**Table 4:** Non-Pharmacologic and Procedural Treatments for Neuropathic Pain.

Intervention	Description	Mechanism of Action	Applications	Risks/Limitations
**Spinal Cord Stimulation (SCS)** [22,23]	Epidural implantation of electrodes	Activates large-diameter Aβ fibers to inhibit nociceptive C-fibers	CRPS, failed back surgery syndrome, peripheral neuropathy	Lead migration, infection, neurological injury, efficacy loss over time
**Transcutaneous Electrical Nerve Stimulation (TENS)** [21]	Non-invasive electrical stimulation via surface electrodes	Stimulates Aβ fibers to inhibit C-fiber input, reduces central sensitization	Localized pain	Limited evidence for long-term efficacy
**Other Interventions**	Intrathecal drug delivery, surgical decompression	Various	Refractory cases	Invasive procedures with associated risks

## Data Availability

No new data were created or analyzed in this study.
